# Ischemic Retinal Vasculitis Associated with Cataract Surgery and Intracameral Vancomycin

**DOI:** 10.1155/2015/683194

**Published:** 2015-11-05

**Authors:** Lucas T. Lenci, Eric K. Chin, Christi Carter, Stephen R. Russell, David R. P. Almeida

**Affiliations:** ^1^Department of Ophthalmology & Visual Sciences, University of Iowa Hospitals & Clinics, Iowa City, IA 52240, USA; ^2^Vitreoretinal Surgery, PA, 7760 France Avenue S., Minneapolis, MN 55435, USA

## Abstract

Recently, there have been reports suggesting that intracameral vancomycin has been associated with retinal vasculitis; some have described this phenomenon as postoperative hemorrhagic occlusive retinal vasculitis. We present a case of a 65-year-old woman who underwent uncomplicated phacoemulsification and posterior chamber intraocular lens implantation followed by intracameral antibiotic prophylaxis. Unlike prior reports, this report demonstrates a case of mild visual reduction and minimal inflammation with subtle but complete unilateral peripheral retinal ischemia associated with cataract surgery and intracameral vancomycin, suggesting a spectrum of toxicity that may be underrecognized.

## 1. Introduction

Cataract surgery may be associated with postoperative inflammatory or infectious complications, such as toxic anterior segment syndrome (TASS) or endophthalmitis. Typically, TASS or endophthalmitis is heralded by a disproportionate reduction in vision or increase in inflammation. Prior cases reported to be retinal vasculitis associated with cataract surgery and intracameral vancomycin injection showed similar reduction in vision with marked inflammation [1, 2]. In this report, we present a case of mild visual reduction and minimal inflammation with subtle but complete unilateral peripheral retinal ischemia associated with cataract surgery and intracameral vancomycin, suggesting a spectrum of toxicity that may be underrecognized.

## 2. Case Report

**Figure 1:**
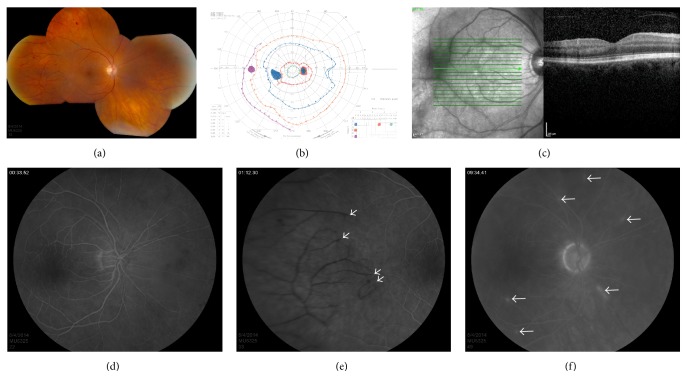
Presentation of ischemic hemorrhagic retinal vasculitis after cataract surgery with intracameral vancomycin. Color fundus montage photograph displays attenuated retinal vessels with arteriolar nonperfusion and distal branch retinal artery occlusions (a). Goldmann visual field shows enlargement of the blind spot to the I4e isopter, paracentral scotoma to the I4e isopter, and central constriction of the I1e and I2e isopters (b). Optical coherence tomography illustrates adequate representation of all retinal layers (c). Fluorescein angiography with delayed retinal and choroidal filling in the mid frame (d). Late frames show numerous peripheral branch retinal artery occlusions (e) and scattered areas of vessel wall hyperfluorescence (f). The left eye was normal with no abnormal findings except moderate nuclear sclerosis.

A 65-year-old woman with a past medical history of hypertension underwent uncomplicated phacoemulsification cataract extraction with implantation of a posterior chamber intraocular lens (PCIOL) in her right eye (OD) that concluded with injection of intracameral vancomycin (Hospira, NDC# 00409-4332-01, Lot# 396158E02, expiration 3/1/2016, concentration 1.0 mg/0.1 cc) as routine infection prophylaxis. Ofloxacin and prednisolone acetate 1% were postoperatively prescribed. On postoperative day 1, her vision was 20/20; however, this decreased two days later to 20/400. There was a 0.9–1.2 log unit relative afferent papillary defect (RAPD) OD. Intraocular pressure (IOP) was 18 mmHg OD. Slit-lamp examination of the right eye revealed mild conjunctival injection, trace corneal edema, deep anterior chamber with trace cell and flare but no hypopyon or fibrin, and a PCIOL in the capsular bag with no pigment or inflammatory deposits on the lens. The vitreous was quiet with no cells. On dilated examination, the optic nerve was normal with no disc edema or disc hemorrhage and a cup-to-disc ratio of 0.3. The retinal vessels were attenuated with box-carring and areas of arteriolar nonperfusion with distal branch retinal artery occlusions and scattered dot-blot hemorrhages in the periphery (Figure 1(a)). The left eye was normal with no abnormal findings except moderate nuclear sclerosis. The patient was cognizant of a paracentral scotoma that was confirmed by Goldmann visual field (GVF) (Figure 1(b)). Optical coherence tomography (OCT) showed preservation of all posterior retinal layers (Figure 1(c)). Fluorescein angiogram showed delayed retinal and choroidal filling with numerous peripheral branch retinal artery occlusions and scattered areas of vessel wall hyperfluorescence (Figures 1(d)–1(f)). Wide-field montages of mid- and late-phase angiograms showed the border of ischemia to be nearly equidistant about the posterior pole.

**Figure 2:**
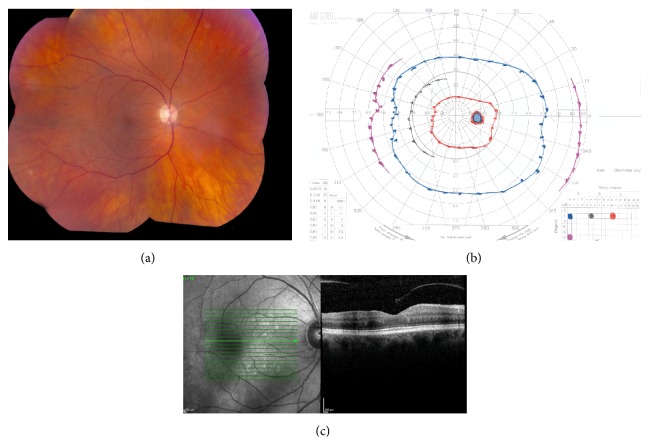
Resolution of ischemic retinal vasculitis after eight weeks. Color fundus montage photograph shows posterior pole arteriolar sheathing with attenuation in the retinal periphery (a). Goldmann visual field shows resolution of previous visual field defects (b). Optical coherence tomography is stable compared to previous with adequate representation of all retinal layers (c). The left eye was never affected.

Given the evidence of retinal ischemic vasculitis and lack of signs of endophthalmitis or TASS, the patient was observed closely maintaining her ofloxacin and prednisolone drops and an inflammatory workup initiated. The procedure was uncomplicated and the patient did not have any hypertensive episodes. Her blood pressure was 150/67 and she continued being on her normal antihypertensive regimen. On postoperative day 4, her vision improved to 20/40 with unchanged mild inflammation. On day 5, her vision further improved to 20/25 OD and, at this time, the anterior chamber was quiet and the pupillary response partially normalized to a 0.3–0.6 log unit RAPD. Posterior segment examination revealed stable attenuation of retinal vessels with areas of arteriolar nonperfusion. Two months later, her vision returned to 20/20 with no RAPD (<0.3 log units). The right eye showed arteriolar sheathing and peripheral attenuation with collateral vessel formation in the temporal mid periphery (Figure 2(a)); GVF scotoma had resolved (Figure 2(b)). The left eye was normal and unchanged. The patient had no prior history of hypercoagulability or thromboses. All systemic investigations were negative including complete blood count, erythrocyte sedimentation rate, C-reactive protein, syphilis serology, tuberculosis quantiferon gold, anti-nuclear antibody, rheumatoid factor, and anti-neutrophil cytoplasmic antibodies.

## 3. Discussion

We present a case of ischemic retinal vasculitis associated with intraoperative intracameral vancomycin during cataract surgery. In the literature, there is one report of two cases of retinal vasculitis after cataract surgery with vancomycin and one more recent case series which described this sequela as postoperative hemorrhagic occlusive retinal vasculitis [1, 2]. In contrast to the prior reported cases that had severe anterior chamber and vitreous inflammatory reactions with profound irreversible vision loss, our case had minimal inflammatory reaction and significant retinal vasculitis with multiple arterial occlusions in a circular distribution about the posterior pole. The present case demonstrated rapid resolution when treated with a short course of topical medications.

Although it is impossible to confirm the relationship of this disease to vancomycin exposure, we believe that it is unlikely to be related to an underlying systemic process or autoimmune disease. The patient had no history of hypertensive episodes or history of thrombosis. This contention is also supported by the abrupt onset, rapid resolution, temporal and ipsilateral association to vancomycin and the lack of prior symptoms or signs of uveitis, and negative laboratory workup. Although we believe the etiology is unlikely to be TASS (there were no other affected cases at the same surgery center on that day and our case had minimal anterior chamber reaction), the most provocative finding was the rapid normalization of the afferent pupillary defect and vision.

Vancomycin has been recommended for widespread prophylactic use in cataract surgery [3, 4]. Vancomycin is also widely used as an intravitreal injection for endophthalmitis [5]. Vancomycin retinal toxicity has been demonstrated in rabbits receiving intravitreal injections of vancomycin in silicone-filled eyes but human toxicity has not been suggested until recently [1, 6]. If this case represents the mild end of a spectrum of vancomycin toxicity, we believe that prospective evaluation will be needed to determine the incidence of the transitory and variable nature of the visual impact of widespread vancomycin surgical prophylaxis.

## References

[1] Nicholson L. B., Kim B. T., Jardón J. (2014). Severe bilateral ischemic retinal vasculitis following cataract surgery. *Ophthalmic Surgery, Lasers and Imaging Retina*.

[2] Witkin A. J., Shah A. R., Engstrom R. E. (2015). Postoperative hemorrhagic occlusive retinal vasculitis: expanding the clinical spectrum and possible association with vancomycin. *Ophthalmology*.

[3] Anijeet D. R., Palimar P., Peckar C. O. (2010). Intracameral vancomycin following cataract surgery: an eleven-year study. *Clinical Ophthalmology*.

[4] Gore D. M., Angunawela R. I., Little B. C. (2009). United Kingdom survey of antibiotic prophylaxis practice after publication of the ESCRS Endophthalmitis Study. *Journal of Cataract and Refractive Surgery*.

[5] Lemley C. A., Han D. P. (2007). Endophthalmitis: a review of current evaluation and management. *Retina*.

[6] Hegazy H. M., Kivilcim M., Peyman G. A. (1999). Evaluation of toxicity of intravitreal ceftazidime, vancomycin, and ganciclovir in a silicone oil-filled eye. *Retina*.

